# The MEK1/2 inhibitor, selumetinib (AZD6244; ARRY-142886), enhances anti-tumour efficacy when combined with conventional chemotherapeutic agents in human tumour xenograft models

**DOI:** 10.1038/bjc.2012.8

**Published:** 2012-02-16

**Authors:** S V Holt, A Logié, R Odedra, A Heier, S P Heaton, D Alferez, B R Davies, R W Wilkinson, P D Smith

**Affiliations:** 1 Oncology iMED, AstraZeneca, Alderley Park, Macclesfield SK10 4TG, UK; 2 Clinical and Experimental Pharmacology, Paterson Institute for Cancer Research, Wilmslow Road, Manchester M20 4BX, UK; 3 Safety Assessment, AstraZeneca, Alderley Park, Macclesfield SK10 4TG, UK

**Keywords:** Selumetinib, barasertib, docetaxel, temozolomide, scheduling, apoptosis

## Abstract

**Background:**

The Ras/RAF/MEK/ERK pathway is frequently deregulated in cancer and a number of inhibitors that target this pathway are currently in clinical development. It is likely that clinical testing of these agents will be in combination with standard therapies to harness the apoptotic potential of both the agents. To support this strategy, it has been widely observed that a number of chemotherapeutics stimulate the activation of several intracellular signalling cascades including Ras/RAF/MEK/ERK. The MEK1/2 inhibitor selumetinib has been shown to have anti-tumour activity and induce apoptotic cell death as a monotherapy.

**Methods:**

The aim of this study was to identify agents, which would be likely to offer clinical benefit when combined with selumetinib. Here, we used human tumour xenograft models and assessed the effects combining standard chemotherapeutic agents with selumetinib on tumour growth. In addition, we analysed tumour tissue to determine the mechanistic effects of these combinations.

**Results:**

Combining selumetinib with the DNA-alkylating agent, temozolomide (TMZ), resulted in enhanced tumour growth inhibition compared with monotherapies. Biomarker studies highlighted an increase in *γ*H2A.X suggesting that selumetinib is able to enhance the DNA damage induced by TMZ alone. In several models we observed that continuous exposure to selumetinib in combination with docetaxel results in tumour regression. Scheduling of docetaxel before selumetinib was more beneficial than when selumetinib was dosed before docetaxel and demonstrated a pro-apoptotic phenotype. Similar results were seen when selumetinib was combined with the Aurora B inhibitor barasertib.

**Conclusion:**

The data presented suggests that MEK inhibition in combination with several standard chemotherapeutics or an Aurora B kinase inhibitor is a promising clinical strategy.

Members of the Ras/RAF/MEK/ERK pathway, in particular KRAS and BRAF, are frequently deregulated in several cancers including melanoma, colorectal (CRC), non-small cell lung cancer (NSCLC) and pancreatic (Sebolt-Leopold and Herrera, 2004). Activating mutations in KRAS for example are present in ∼90% of pancreatic cancers, ∼20% of CRC and ∼35% of NSCLC with B-RAF mutations present in ∼20–60% of melanomas, 35–70% of papillary thyroid and ∼12% of CRC (Sebolt-Leopold and Herrera, 2004). Therefore, targeting this pathway is an attractive therapeutic strategy for the treatment of cancer.

The MAPK signalling cascades are activated by several stimuli, including growth factors and hormones, which regulate gene expression and cell survival. Three major subfamilies of the MAPK pathways have been identified; JNK/SAPK's, p38 MAPK and MEK/ERK. Primarily in oncology, several MEK inhibitors have been developed and are undergoing clinical testing (Rinehart *et al*, 2004; Lorusso *et al*, 2005; Banerji *et al*, 2010; Infante *et al*, 2010). MEK contains two consensus kinase motifs, which are involved in the phosphorylation of serine/threonine and tyrosine residues. Two homologues exist, MEK1 and MEK2, which have only one known substrate, ERK1/2, which has multiple downstream effectors involved in a number of cellular functions including transcription (e.g. Elk1), cell cycle progression (e.g. Rb) and cell motility (e.g. JNK) (Sebolt-Leopold and Herrera, 2004). p90RSK is also phosphorylated by ERK1/2 and has a number of substrates including elongation initiation factor (eIF) 4E-binding protein (4E-BP1), ribosomal protein S6 (S6) and tuberin (TSC2), which are also downstream substrates of the mTOR pathway.

Once activated, ERK, along with RAF and MEK, migrate to the nucleus where they activate cyclin D1 and downregulate p27 thus driving cell proliferation (Bhatt *et al*, 2005). Activation of this signalling cascade has been shown to drive entry into G1 but there are also a number of reports, which suggest members of this pathway are required for normal G2/M progression and entry into mitosis (Wright *et al*, 1999; Hayne *et al*, 2000). Activation of the ERK pathway can also inhibit apoptosis by phosphorylating several pro-apoptotic proteins including Bim-EL and Bad (Scheid *et al*, 1999; Biswas and Greene, 2002; She *et al*, 2005). Furthermore, active ERK can enhance the activity of anti-apoptotic proteins including Bcl-2 and Mcl-1 (Deng *et al*, 2000; Domina *et al*, 2004). Inhibition of this pathway should therefore drive pro-apoptotic signalling or reduce the threshold for apoptosis induction by other agents.

Commonly used chemotherapeutics, including DNA-damaging and microtubule-stabilising agents, ultimately function to drive cell death but do require cells to be in cycle for this to occur. Furthermore, these agents have also been shown to promote the activation of several cell survival pathways including MAPK (MacKeigan *et al*, 2002). Clinically, there is a great potential for combinations of MEK inhibitors and conventional chemotherapeutics. However, in order to increase the chance of success of these trials rational combination partner selection, dosing schedules and/or mechanistic understanding of dual therapies must be explored pre-clinically.

In this report, we present our findings demonstrating that combining the MEK1/2 inhibitor, selumetinib, with several standard chemotherapeutic agents resulted in enhanced anti-tumour efficacy in human tumour xenograft models. In order to understand the mechanistic benefits of these combinations, we investigated both the phenotypic and the pharmacodynamic response when selumetinib was combined with either docetaxel or temozolomide (TMZ).

## Materials and methods

### Chemicals

Selumetinib (AZD6244) and barasertib (AZD1152) were prepared as previously reported (Davies *et al*, 2007; Wilkinson *et al*, 2007). TMZ (Schering Corporation, Kenilworth, NJ, USA) was formulated in corn oil and docetaxel (Sanofi-Aventis, Paris, France) was formulated in 2.6% ethanol.

### Cell culture

All cell lines were cultured in L-15, McCoy's or RPMI 1640+10% foetal calf serum (FCS)+1% glutamine or RPMI 1640+20% FCS+1% glutamine+1% sodium pyruvate. All cells were obtained from the American Type Culture Collection.

### *In vivo* studies

Female nude mice (*nu/nu:Alpk*; AstraZeneca, Macclesfield, UK) were housed in negative pressure isolators (PFI Systems Ltd., Milton Keynes, UK). Experiments were conducted in 8–12-week-old animals in full accordance with the United Kingdom Home Office Animal (Scientific Procedures) Act 1986. Human tumour xenografts were established by subcutaneous (s.c.) injection of 1 × 10^7^, 5 × 10^6^, 1 × 10^6^ and 3 × 10^6^ cells per mouse for HCT-116 (CRC), SW-620 (CRC), CaLu-6 (NSCLC) and AsPc-1 (Pancreatic), respectively. Animals were randomised into treatment groups (*n*=8 to 14) when tumours reached a defined palpable size (0.2 or 0.4 cm^3^). Selumetinib and TMZ were administered by oral gavage, docetaxel by intravenous injecton. All compounds were prepared at 0.1 ml per 10 g. Barasertib was prepared in Tris buffer (pH 9) and administerd as a continuous 48 h infusion via s.c. implanted osmotic mini-pumps (2 × 24 h pumps implanted sequentially; model 2001D, Durect Corp., Cupertino, CA, USA). Tumour volume (measured by callipers), animal body weight and tumour condition were recorded twice weekly during the study. Growth inhibition from the start of treatment was assessed by comparison of the differences in tumour volume between control and treated groups. Because the variance in mean tumour volume data increases proportionally with volume (and is therefore disproportionate between groups), data were log transformed to remove any size dependency before statistical evaluation. Statistical significance was evaluated using a one-tailed, two sample *t*-test.

### Pharmacodynamic sampling

At a defined timepoints, selected mice were humanely culled and tumours were excised and either snap-frozen in liquid nitrogen, or fixed in 10% buffered formalin for 24 h before imbedding in paraffin for immunohistochemical (IHC) staining.

### Immnunoblotting

Protein lysates were prepared from frozen tumours using the FastPrep-24 (MP Biomedicals, Solon, OH, USA) in 10 × lysis buffer (#9803; Cell Signaling Technology, Danvers, MA, USA) diluted to 1 × in PBS containing phosphatase inhibitor cocktail I and II (P2850 and P5726; Sigma, St Louis, MO, USA) and protease inhibitor cocktail (P8340; Sigma). Protein concentration was determined by BCA assay (Pierce, Cramlington, UK). Total protein of 20 μg per sample were resolved on 12% Bis-Tris SDS–PAGE gels (Invitrogen), transferred to nitrocellulose membranes (Invitrogen, Grand Island, NY, USA) and incubated with anti-Bim (1 : 1000; Chemicon, Watford, UK), total MAPK (1 : 1000; Cell Signaling Technology), phospho MAPK (Thr202/Tyr204) (1 : 1000; Cell Signaling Technology; and GAPDH (1 : 2000; Abcam, Cambridge, UK) and subsequently horseradish peroxidase-conjugated anti-rabbit or anti-mouse IgG (1 : 2500; Cell Signaling Technology). Immunoreactive proteins were detected by enhanced chemiluminescence (Pierce) and bands were detected and quantitated on a Chemigenius (Syngene, Cambridge, UK).

### Immunohistochemistry

Sections were dewaxed and endogenous peroxidase activity was blocked with 3% (v/v) hydrogen peroxide for 10 min. Heat-mediated antigen retrieval was achieved by incubating the sections in 0.01 M citrate (pH6.0) for 5 min at 110°C in a Milestone Rapid Microwaves Histoprocessor (model RHS-2) to retrieve antigen. Sections were placed in 0.01 ml l^–1^ citrate (pH 6) and incubated for 5 min at 110°C. Sections were cooled and transferred to a Lab Vision Autostainer. Following incubation with Dako serum-free protein block (Dako, Ely, UK, XO909) for 20 min, sections were incubated with anti-phospho-Histone H3, pHH3 (Ser10), (Upstate BioTechnology, Watford, UK, #06-570), Phospho-Histone Histone H2A.X, *γ*H2A.X (ser139), (Cat # 2577) and Cleaved Caspase-3 (CC3) (Asp175), (Cat #9661) (all antibodies purchased from Cell Signaling Technologies) antibodies at room temperature for 60 min. Sections were subsequently incubated with mouse/rabbit-labelled polymer from mouse EnVision kit (Dako) for 30 min at room temperature, and signal was detected using the En Vision kit 3.3′-diamino benzidine (Dako, K3468). Sections were counterstained with Carazzi's haematoxylin and mounted in Histomount (Fisher, Loughborough, UK). Cleaved caspase 3 and pHH3-positive cells were scored using algorithms developed for scoring percentage positive nuclei on an ACIS II image analyzer (ChromaVision Medical Systems, Inc., San Juan, Capistrano, CA, USA) using standard threshold settings for each marker. Representative images were taken using an Aperio Image Scope 10.2 using the × 20 objective and a subsequent zoom of × 20.

### Flow cytometry

Tumour tissues snap frozen in liquid nitrogen were disaggregated processed for propidium iodide staining (Sigma) as previously described (Wilkinson *et al*, 2007). Quantitation of polyploidy and sub G1 cells was performed using ModFit LT (Verity Software House, Topham, ME, USA).

## Results

### Combined treatment of selumetinib and standard of care agents results in enhanced anti-tumour efficacy

**Figure 1:**
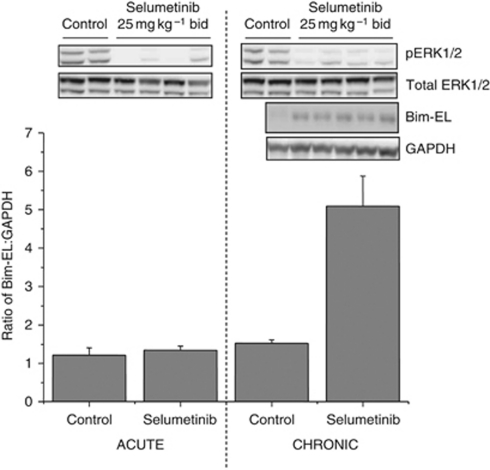
A chronic dosing schedule of selumetinib increases levels of the pro-apoptotic protein Bim in HCT-116 xenografts. When tumours reached an average volume of 0.2 cm^3^, animals (*n*=5 per group) were dosed with either 25 mg kg^–1^ per bid of selumetinib or vehicle control. Following treatment, tumours were excised after either 3 (acute) or 14 (chronic) doses and analysed for pharmacodynamic effects. Immunoblotting analysis of lysed tumour tissue was used to detect pERK1/2, total ERK1/2 and Bim-EL. Bim-EL levels were calculated in a ratio to GAPDH. The bar graph shows the average across the group ±s.e.m.

Mechanistic biomarker studies of the effects of selumetinib in human tumour xenograft models have shown that, in addition to pERK1/2 downregulation, a sustained exposure to the agent results in an increase in downstream apoptotic signalling and a decrease in cell cycle progression (Davies *et al*, 2007). Furthermore, a chronic dosing schedule of selumetinib (25 mg kg^–1^ per bid for 14 doses) in HCT-116 xenografts increased the levels of the pro-apoptotic BH3 protein Bim-EL (∼4-fold increase) compared with no change with the level of this protein following a shorter dosing period (three doses) (Figure 1). In order to exploit the apoptotic threshold of selumetinib, we wanted to study the effects of combining it with agents known to induce the apoptotic cascade to drive tumour growth inhibition and/or cell death.

The effects of selumetinib in combination with a number of key standard of care agents were tested pre-clinically in human tumour xenograft models and resulted in enhanced anti-tumour activity. A number of compounds including the DNA-alkylating agent TMZ and the anti-mitotic drug docetaxel resulted in a greatly enhanced tumour growth inhibition compared with monotherapies (Table 1 and supplementary Table 1). However, combining selumetinib with gemcitabine did not enhance the anti-tumour activity of the individual agents (supplementary Table 1). The data presented here suggests that when selumetinib is combined, with either TMZ or docetaxel, the resulting anti-tumour phenotypes maybe due to mechanistic interactions between the two compounds.

### Selumetinib in combination with TMZ enhances DNA damage

**Figure 2:**
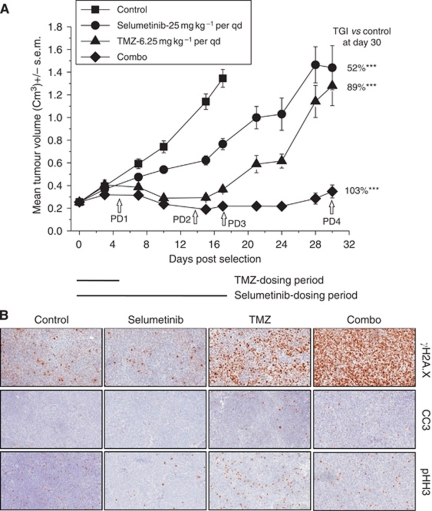
Selumetinib in combination with TMZ enhances DNA damage. When female nude mice bearing SW-620 human tumour xenografts reached an average volume of 0.2 cm^3^, animals were randomised (*n*=10 for controls and *n*=8 per treatment groups) and dosed with either selumetinib (25 mg kg^–1^ per qd), TMZ 6.25 mg kg^–1^ for 5 consecutive days, selumetinib+TMZ or vehicle controls. A subsequent study was performed in order to generate pharmacodynamic (PD) samples (*n*=5 per group) following exposure to the dosing regimens at the PD points highlighted by the white arrows on the (**A**) tumour growth inhibition graph; (**B**) representative IHC images for *γ*H2A.X, cleaved caspase 3 and pHH3 at PD2. All error bars are ±s.e.m. ^***^P<0.0005.

The combination of selumetinib and TMZ in the SW-620 human tumour xenograft model resulted in a significantly enhanced anti-tumour efficacy (103.5% inhibition; *P*<0.0005), compared with selumetinib (52% inhibition; *P*<0.0005) and TMZ (88% inhibition; *P*<0.0005) alone (Figure 2A). At the end of the dosing period, the monotherapy and combination treatment groups were left to observe the growth rate following the cessation of dosing (animals in the control group had to be killed due to tumour size). In the selumetinib and TMZ monotherapy-treated groups, tumour growth progressed rapidly once compound treatment had been removed. In contrast, the start of tumour growth in the combination group was delayed for approximately 24 days from the start of the experiment compared with 15 days in the TMZ alone group.

In order to investigate potential mechanisms, to explain the enhanced combination effect with TMZ, we used the growth inhibition data generated from our anti-tumour studies to guide our pharmacodynamic sampling times (Figure 2A). Samples were collected at the end of the TMZ dosing (PD1), when the TMZ and combination groups growth rate started to diverge (PD2), at the end of selumetinib dosing (PD3) and at the end of the re-growth period (PD4) (Figure 2A; white arrows on graph). IHC analysis and histological scoring was performed on all the tissues collected to examine, selumetinib effects (pERK1/2), DNA damage (*γ*H2A.X), apoptosis (cleaved caspase 3) and the cell cycle (pHH3) (scoring data not shown). The sampling timepoint, which gave us the greatest insight into the mechanistic effects of these agents was that taken when we started to see the TMZ and combination groups diverge (PD2). As expected, in response to selumetinib or TMZ alone we saw changes in the mechanistic biomarkers pERK1/2 (decrease) and *γ*H2A.X (increase), respectively, and a reduction in mitotic cells as shown by pHH3 in the selumetinib group compared with an increase in the TMZ tissues (Figure 2B). Level of the apoptotic marker, cleaved caspase 3, was comparable in the combination group compared with the TMZ monotherapy (Figure 2B). However, in the combination group we observed a greatly enhanced upregulation of *γ*H2A.X compared with the TMZ group alone, suggesting that when selumetinib and TMZ are combined DNA damage is enhanced (Figure 2B).

### Selumetinib is efficacious in combination with docetaxel when dosed concurrently or following docetaxel

**Figure 3:**
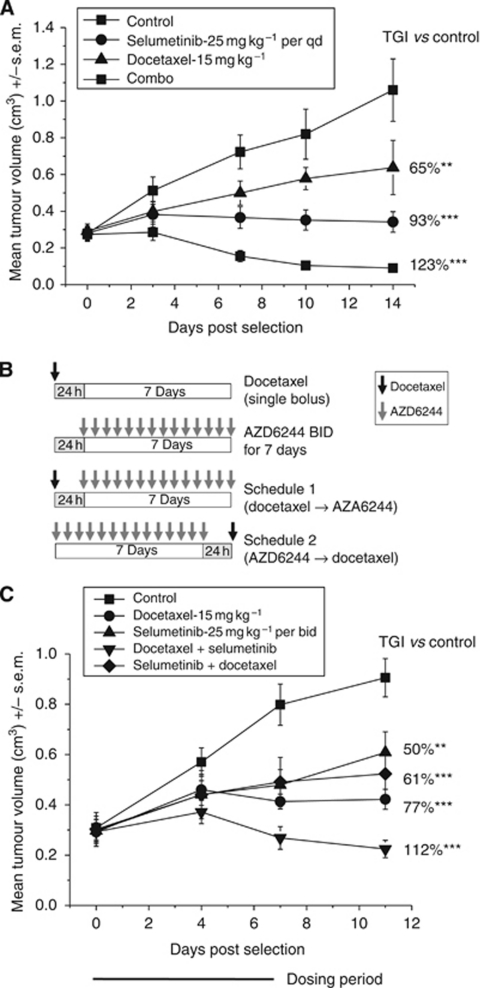
The sequence dependency of selumetinib when combined with docetaxel enhances apoptotic cell death. When female nude mice bearing HCT-116 human tumour xenografts reached an average volume of 0.2 cm^3^, animals were randomised (*n*=12 for controls and *n*=8 per treatment groups) and dosed with either (**A**) selumetinib (25 mg kg^–1^ per qd), docetaxel 15 mg kg^–1^ once weekly or both agents in continuous combination for 14 days or (**B**) following the sequence schedules shown and administered in (**C**) for one 7-day cycle. All error bars are ±s.e.m. ^**^P<0.005, ^***^P<0.0005.

Continuous, concurrent combinations of selumetinib and docetaxel resulted in significant anti-tumour effects in several models (Table 1 and Figure 3A). However, we were interested to explore the effect of dose sequencing on the anti-tumour efficacy of these two agents as administration of selumetinib and docetaxel as monotherapies results in distinct cell cycle phenotypes; a G1 or mitotic arrest, respectively. Two schedules were designed in which mice bearing HCT-116 CRC tumours were treated with either a single dose of docetaxel (15 mg kg^–1^) followed 24 h later by selumetinib (25 mg kg^–1^ per bid) for 7 days (schedule 1) or selumetinib was administered as above for 7 days followed 24 h later with docetaxel (schedule 2) (Figure 3B). As monotherapies, docetaxel and selumetinib resulted in 77% (*P*<0.0005) and 50% (*P*<0.005) tumour growth inhibition, respectively (Figure 3C). Interestingly, in the two combination schedules docetaxel administered before selumetinib (schedule 1) resulted in a 110% (*P*<0.0005) compared with 61% (*P*<0.005) tumour growth inhibition when docetaxel was administered after selumetinib (schedule 2). These results suggest that selumetinib could enhance the efficacy of docetaxel when administered in the sequence of docetaxel followed by selumetinib.

### The sequence dependency of selumetinib when combined with docetaxel enhances apoptotic cell death

**Figure 4:**
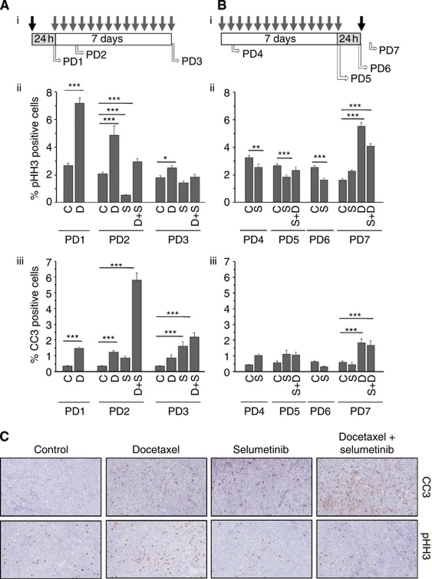
The sequence dependency of selumetinib when combined with docetaxel enhances apoptotic cell death. Following the dosing schedules in Figure 3B, pharmacodynamic (PD) samples (*n*=4 per group) were taken at various points during the dosing cycles (PD1-7) as shown on the schedules in (**A** and **B**). Samples from both schedules were analysed by IHC for pHH3 and cleaved caspase 3 (**C**) representative IHC images for PD2 to illustrate the quantitation shown in (**A**). Abbreviations: C=control, D=docetaxel; S=selumetinib. All graphs represent the average data for each sample set ±s.e.m. ^***^P<0.0005, ^**^P<0.005, ^*^P<0.05.

In order to determine the mechanism of action in the scheduling studies with docetaxel, a series of tumour tissue samples were taken at different time points following exposure to the two dosing regimens (Figure 4Ai and Bi). To assess the cell cycle mechanistic effects the mitotic marker pHH3 was quantitated. In the sequence when docetaxel was administered before selumetinib, increased levels of pHH3 were observed at the early timepoints (PD1 & PD2) (2.7- and 2.3-fold change, respectively; *P*<0.0005). In schedule 2, pHH3 levels were seen to increase following docetaxel (PD7) (3.4-fold; *P*<0.0005) (Figure 4Aii and 4Bii). When selumetinib was administered alone levels of pHH3 decreased at several measurement points compared with controls (PD2 *P*<0.0005; PD4 *P*<0.005; PD5 *P*<0.0005; PD6 *P*<0.0005) consistent with the inhibition of the MEK/ERK pathway resulting in a G1/S arrest. Interestingly, in both sequences in the combination there is a reduction in pHH3 compared with the docetaxel alone group at that timepoint (PD2 and PD7) (3.8- and 2.5-fold change, respectively; *P*<0.0005).

Levels of the apoptotic marker cleaved caspase 3, in tumour tissue taken from the docetaxel followed by selumetinib group, increased in this combination group (16.8-fold change from the control; *P*<0.0005) compared with the single agents alone (3.5- and 2.4-fold change for docetaxel and selumetinib, respectively) (Figure 4Aiii PD2). In comparison, tumour tissue analysed in the same study after the chronic dosing schedule of selumetinib did not demonstrate an increase in cleaved caspase 3 levels in the combination group when compared with the single agents alone. When selumetinib was dosed before docetaxel an increase in cleaved caspase 3 was not observed in the combination group at any of the sampling timepoints compared with at least one of the monotherapies (Figure 4Biii). Representative IHC images from PD2 highlights the increase in pHH3 following docetaxel exposure and the increase in cleaved caspase 3 in the docetaxel followed by selumetinib group (Figure 4C). The data presented here suggests that the combination efficacy effect seen when docetaxel was dosed before selumetinib was due to an increase in apoptosis.

### Sequence scheduling of selumetinib and the Aurora B kinase inhibitor, barasertib (AZD1152), results in tumour regression and increased cell death

**Figure 5:**
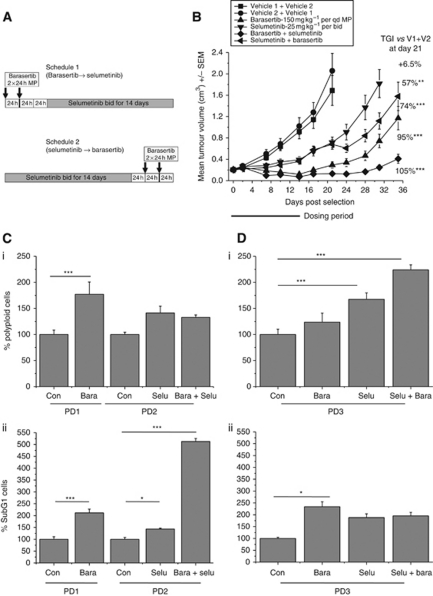
Sequence scheduling of selumetinib and the Aurora B kinase inhibitor, barasertib (AZD1152), results in tumour regression and increased cell death. (**A**) Two dosing schedules investigated in which barasertib was administered at 150 mg kg^–1^ by mini-pump in 2 × 24-h infusions followed by a 24-h break and then selumetinib 25 mg kg^–1^ per bid for 14 days or the reversal of this schedule, (**B**) when CaLu-6 human tumour xenografts reached an average of 0.2 cm^3^ animals were randomised into the relevant vehicle treated, monotherapy or combination groups and exposed to the sequence scheduling. A subsequent study was performed in order to generate pharmacodynamic (PD) samples (*n*=4 per group) at the timepoints, PD 1-3, highlighted in (**A**). (**C** and **D**) flow cytometric analysis was performed on disaggregated tumours in order to investigate levels of polyploidy and sub G1. Abbreviations: Con=control, Bara=barasertib; Selu=selumetinib. All bar graphs represent the average for each group ±s.e.m. ^*^P<0.05, ^**^P<0.005, ^***^P<0.0005.

The dose-scheduling effects of combining selumetinib and docetaxel lead us to investigate the sequence dependency of selumetinib combined with another mitotic targeting agent, the Aurora B kinase inhibitor, barasertib (AZD1152) (16). We designed two regimes in which barasertib was dosed at 150 mg kg^–1^ per qd for 2 consecutive days through a mini-pump (MP) with a 24 h gap followed by selumetinib 25 mg kg^–1^ per bid for 14 consecutive days (schedule 1) or the reverse of this schedule where barasertib was dosed following selumetinib treatment (schedule 2) (Figure 5A). The CaLu-6 NSCLC human tumour xenograft model was used in this study as previous experience with both agents allowed us to select appropriate dose levels in order to investigate these schedules (Davies *et al*, 2007; Wilkinson *et al*, 2007). Selumetinib and barasertib alone resulted in 57% (*P*<0.005) and 95% (*P*<0.0005) tumour growth inhibition compared with the vehicle-treated controls at day 21 after the start of dosing. In comparison when selumetinib was dosed before barasertib the anti-tumour efficacy was 74% (*P*<0.0005) in contrast to 106% (*P*<0.0005) observed when selumetinib was dosed following barasertib. At 21 days after the start of dosing the control, animals had to be culled due to tumour size; however, the monotherapy and combination-treated groups were kept on study for a further 14 days. During this time, the tumours in the monotherapy and schedule 2 groups started to re-grow. Interestingly, the tumours in the group where barasertib was administered before selumetinib (schedule 1) tumour re-growth was delayed (Figure 5B).

In order to investigate this further, we performed pharmacodynamic analysis on tumour tissue. In schedule 1 we analysed tumour tissue at 24 h after the end of the barasertib infusion (PD1) and at the end of the selumetinib-dosing period (PD2) (Figure 5A). In schedule 2, tumours were harvested 24 h after the end of the barasertib infusion at the end of the study (PD3) (Figure 5A). Using flow cytometry we assessed tissues for polyploidy and demonstrated that compared with the vehicle-treated control group, barasertib-treated tumours resulted in increased polyploidy (1.7-fold change; *P*<0.05) in the PD1 samples consistent with the mechanism of this agent (Figure 5C) (Wilkinson *et al*, 2007).

In the same experiment, we monitored the population of sub G1 cells in these groups. At the end of the dosing period in schedule 1 (PD2), there was a significant increase (3.5-fold change; *P*<0.0005) in the sub G1 population in the combination compared with the vehicle-treated controls and selumetinib monotherapy (Figure 5Cii). In comparison, the sub G1 populations in schedule 2 (PD3) were increased ∼2-fold in both the monotherapy and combination groups (Figure 5Dii). These results suggest that the sustained anti-tumour effect and regression observed when barasertib is scheduled before selumetinib is likely to be due to an avoidance of cell cycle-mediated antagonism which allowed an increase in cell death.

## Discussion

In this study, we identified a number of conventional chemotherapeutic agents and the Aurora B inhibitor barasertib, which, when combined with the MEK inhibitor selumetinib, resulted in enhanced efficacy in human tumour xenograft models. In order to try and understand pharmacodynamically why these combinations were more efficacious than single agents alone, we performed biomarker studies with selumetinib and two of its most effective combination partners, TMZ and docetaxel. In addition to this, we also investigated the sequence scheduling of selumetinib and docetaxel and further demonstrated using the Aurora B inhibitor, barasertib, that the sequence scheduling of selumetinib and agents targeting mitosis may warrant consideration clinically in order to achieve optimal therapeutic benefit.

A number of studies have previously described that combining inhibitors of the Ras/RAF/MEK/ERK cascade with the microtubule stabilising agent docetaxel (or paclitaxel) results in an enhanced anti-tumourigenic phenotype (McDaid and Horwitz, 2001; Yu *et al*, 2001; MacKeigan *et al*, 2002; Yacoub *et al*, 2003; McDaid *et al*, 2005; Haass *et al*, 2008). The rationale for these observations is that taxanes have been observed to increase signalling through the MEK/ERK pathway, although the generic effect of this has been debated (Yu *et al*, 2001). Indeed, it has been suggested that only those cell lines which have high levels of endogenous MEK/ERK pathway activation are those in which synergy is seen when a taxane and MEK inhibitor are combined (McDaid and Horwitz, 2001). Work presented here, and previously from our laboratory (Davies *et al*, 2007), has shown that in human tumour xenograft models a continuous combination of selumetinib and docetaxel resulted in enhanced anti-tumour efficacy compared with monotherapies.

Although concurrent dosing of selumetinib and docetaxel is highly effective in pre-clinical models (Haass *et al*, 2008) in view of the role of MAPK in the cell cycle and in particular involvement in mitotic entry/progression, we wanted to understand whether there was any benefit from the scheduling order of selumetinib with docetaxel dosing. In this study, we set up two dosing schedules one in which docetaxel was administered 24 h before selumetinib and the other where docetaxel was dosed 24 h after the end of the selumetinib treatment schedule. Our results showed that selumetinib dosed following exposure to docetaxel is the preferred sequence of administration.

The effects of sequence scheduling have been previously demonstrated *in vitro* when taxanes and MEK inhibitors were combined, suggesting administration of the taxane followed by MEK inhibition was more beneficial (Yu *et al*, 2001; Yacoub *et al*, 2003). Our subsequent pharmacodynamic studies demonstrated that in the schedule where docetaxel was administered first we saw enhanced cleaved caspase 3 signalling, suggesting that the enhanced efficacy was due to enhanced apoptotic signalling. This further supports investigations in cell lines in which paclitaxel dosing before another MEK inhibitor (PD98059) showed enhanced mitochondrial dysfunction, caspase activation and PARP cleavage (Yu *et al*, 2001). Yu *et al* (2001) further described how pre-treatment with paclitaxel induced perturbation in MAPK and p38 signalling pathways that lowered the threshold for mitochondrial injury before addition of the MEK inhibitor. The combination of taxanes and MEK inhibition may also induce the apoptotic cascade by preventing phosphorylation of the pro-apoptotic protein Bim, and thus promoting its accumulation and ability to bind and inhibit the anti-apoptotic proteins Mcl-1 and Bcl-xL (Biswas and Greene, 2002; Ley *et al*, 2003; Luciano *et al*, 2003; Tan *et al*, 2005; Ewings *et al*, 2007). In response to a prolonged exposure to selumetinib, we have observed increased Bim-EL levels in our xenograft tissues. We believe that this could be one of the key mechanistic factors contributing to the enhanced anti-tumour efficacy we observe when selumetinib is combined with docetaxel using a concurrent schedule. Interestingly, this sequence scheduling of prior taxane dosing, has also been demonstrated for combinations with agents targeting the PI3K/AKT pathway when combined with docetaxel (MacKeigan *et al*, 2002; Yacoub *et al*, 2003; Hirai *et al*, 2010). Furthermore, paclitaxel followed by MEK inhibition results in inactivation of AKT and downregulation of PI3K (MacKeigan *et al*, 2002).

Our observations and those of others have presented pre-clinical evidence that combining MEK inhibitors and taxanes is a favourable combination approach moving forward into clinical trials. In addition, there may also be an advantage of sequence-scheduling approaches. However, we wanted to explore whether the benefit of combining MEK inhibitors was restricted to taxanes and other microtubule-stabilising agents (Yacoub *et al*, 2003) or whether any agents targeting mitosis would have combination benefits and whether sequence scheduling is important. ERK1/2 has been shown to have a role not just in microtubule function but also in G2/M progression and the mitotic spindle checkpoint (Liu *et al*, 2004; Zhao and Chen, 2006). In order to explore the effect of combining MEK inhibition and scheduling with other agents targeting mitosis, we investigated the combination of selumetinib with the selective Aurora B inhibitor barasertib. As observed with docetaxel, administering the agent targeting the mitotic axis before selumetinib resulted in a sustained tumour growth inhibition. Our *in vivo* pharmacological investigations also demonstrated that in the group where the Aurora B inhibitor was administered before MEK inhibition, we saw an increase in cell death compared with selumetinib when dosed before barasertib.

The scheduling sequence dependency observed, when mitotic agents and MEK inhibitors are combined, could be due to inhibition of the ERK1/2 pathway resulting in a G1 arrest. Therefore, inhibiting MEK prevents cells entering mitosis and consequently the requirement for agents, such as microtubule targeting agents and Aurora B inhibitors, to target cells in this phase of the cell cycle would not be achieved (Wright *et al*, 1999; Hayne *et al*, 2000; Yan *et al*, 2007). In addition to entry into mitosis, the role of the MAPK pathway in spindle checkpoint activation must also be considered. The spindle checkpoint regulator Mps1 has been shown to be a target of ERK (Zhao and Chen, 2006) and in response to B-RAF^V600E^ signalling potentiates the spindle checkpoint by stabilising Mps1 (Cui and Guadagno, 2008).

In addition to mitotic-targeting agents, we also investigated the benefit of combining selumetinib with the DNA-alkylating agent TMZ/DTIC, a standard of care treatment for melanoma patients. In our studies we used TMZ, as opposed to the registered agent DTIC, as it has more reproducible pharmacokinetics and does not require enzymatic conversion in the liver (Friedman *et al*, 2000). In our studies we saw that combining selumetinib and TMZ resulted in sustained anti-tumour activity. Furthermore, pharmacodynamics demonstrated that in the combination group a sustained increase in *γ*H2A.X was observed, suggesting inhibiting MEK-potentiated DNA damage or inhibited its repair. Raf/MEK/ERK signalling has been shown to be required for the positive and negative regulation of homologous recombination repair (HRR). ATM-dependent signalling through the MEK/ERK pathway is critical for efficient HRR and for radiation-induced ATM activation and is suggestive of a regulatory feedback loop between ERK and ATM (Golding *et al*, 2007). Therefore, if MEK is inhibited the HRR pathway is unable to function and enhanced/sustained DNA damage would be expected. Our observations and those of others highlight the potential clinical benefit of MEK inhibitors in combination with conventional agents tageting DNA damage. However, it is also worth considering that novel agents targeting the DNA damage pathway may offer enhanced anti-tumour activity when combined with MEK inhibition. For example, the Chk1 inhibitor, UCN-01 activates the ERK1/2 pathway. When Chk1 and Ras/RAF/MEK/ERK pathway inhibitors were combined, anti-tumour efficacy and increased DNA damage was observed suggesting a functional role of the Ras/RAF/MEK/ERK signalling in the regulation of Chk1 inhibitor-mediated DNA damage (Dai *et al*, 2008; Pei *et al*, 2011).

In summary, concurrent combination of the MEK1/2 inhibitor selumetinib with a number of conventional chemotherapeutic agents, or barasertib, results in enhanced anti-tumour efficacy in human tumour xenograft models. The role of Ras/RAF/MEK/ERK cascade in key cellular events such as the cell cycle, apoptosis and DNA damage, highlights the potential of combining MEK inhibitors with chemotherapeutics belonging to several functional classes. The work presented here has been able to link the enhanced anti-tumour effects seen when selumetinib is combined with TMZ or docetaxel to enhanced DNA damage and apoptosis, respectively. Furthermore, our studies have also explored the rationale for sequence scheduling when MEK inhibitors and agents targeting mitosis are combined. The work presented in this study does offer a greater mechanistic understanding of these combinations to help support clinical trial designs. Overall, our pre-clinical observations demonstrate mechanistic rationale that combinations of selumetinib with several standard chemotherapeutics or an Aurora B inhibitor may offer clinical benefit.

## Figures and Tables

**Table 1:** Overview of selumetinib and standard of care agents in CRC human tumour xenograft models

**Xenograft model**	**Selumetinib**	**Selumetinib %TGI**	**Cytotoxic dosing schedule per cycle**	**Cytotoxic %TGI**	**No. of cycles**	**Treatment duration (days)**	**Combination %TGI**
SW-620	25 mg kg^–1^ per qd (p.o)	52^***^	Temozolomide 6.25 mg kg^–1^ per qd (p.o. first 5 days)	89^***^	1	18	103^***^
HCT-116	25 mg kg^–1^ per bid (p.o)	93^***^	Docetaxel 15 mg kg^–1^ (i.v once weekly)	65^**^	2	14	123^***^
aSW-620	25 mg kg^–1^ per bid (p.o)	74^***^	Docetaxel 15 mg kg^–1^ (i.v once weekly)	12NS	3	20	98^***^

Abbreviations: CRC=colorectal cancer; i.v.=intravenous; NS=non-significant; p.o.=per os; TGI=tumour growth inhibition; ^***^P<0.0005, ^**^P<0.005.

a Previously published in Wilkinson *et al* (2007).
