# Supplementary material for: The MEK1/2 inhibitor, selumetinib (AZD6244; ARRY-142886), enhances anti-tumour efficacy when combined with conventional chemotherapeutic agents in human tumour xenograft models
Source: Br J Cancer. 2012 Feb 16;106(5):858–66. doi: 10.1038/bjc.2012.8 (PMC3305954; doi:10.1038/bjc.2012.8)
Supplement: Supplementary Table [file bjc20128x1.ppt]

## Slide 1
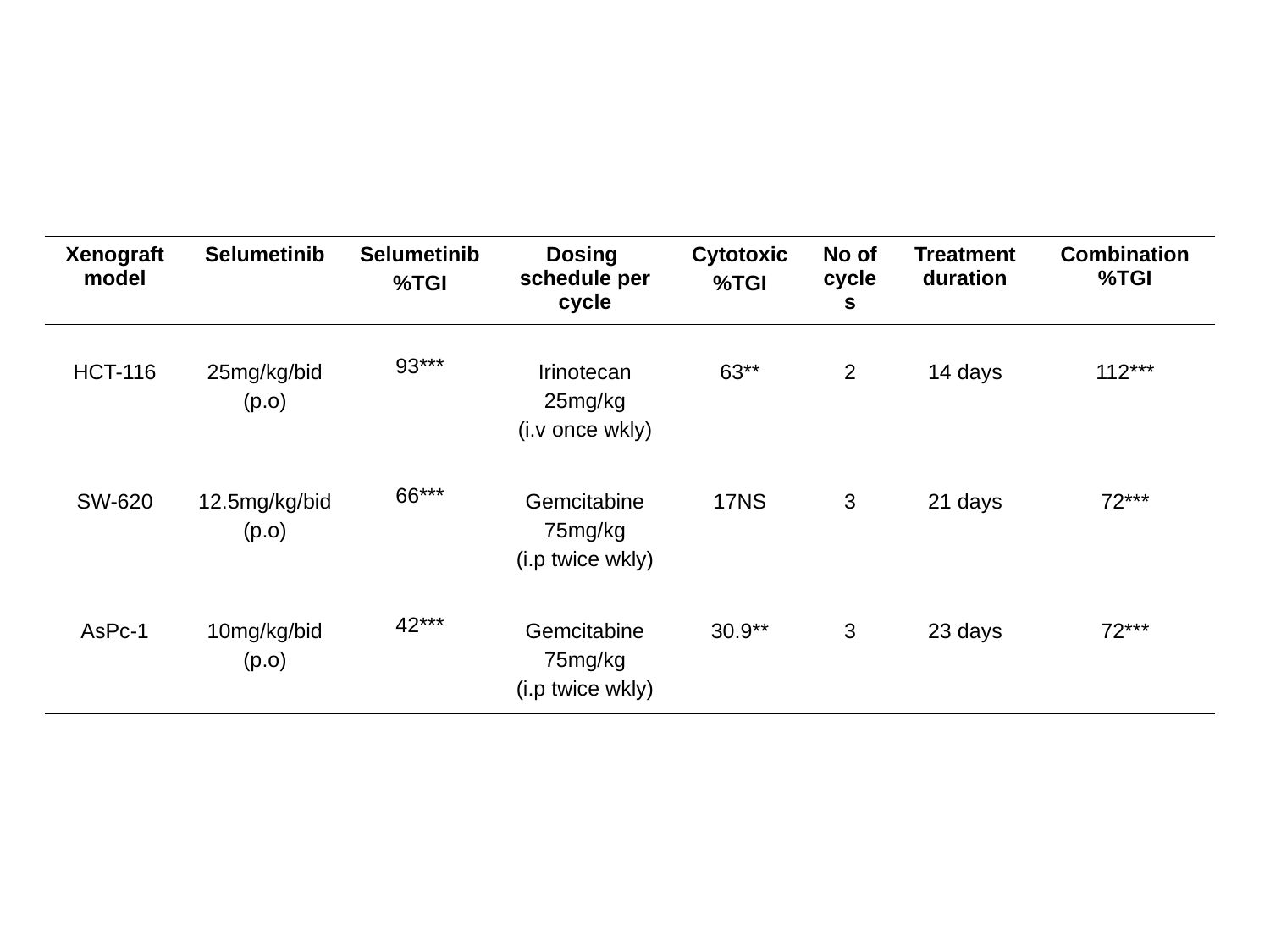

| Xenograft model | Selumetinib | Selumetinib %TGI | Dosing schedule per cycle | Cytotoxic %TGI | No of cycles | Treatment duration | Combination %TGI |
| --- | --- | --- | --- | --- | --- | --- | --- |
| HCT-116 | 25mg/kg/bid (p.o) | 93\*\*\* | Irinotecan 25mg/kg (i.v once wkly) | 63\*\* | 2 | 14 days | 112\*\*\* |
| SW-620 | 12.5mg/kg/bid (p.o) | 66\*\*\* | Gemcitabine 75mg/kg (i.p twice wkly) | 17NS | 3 | 21 days | 72\*\*\* |
| AsPc-1 | 10mg/kg/bid (p.o) | 42\*\*\* | Gemcitabine 75mg/kg (i.p twice wkly) | 30.9\*\* | 3 | 23 days | 72\*\*\* |
